# Rapid oxidative fragmentation of polypropylene with pH control in seawater for preparation of realistic reference microplastics

**DOI:** 10.1038/s41598-023-31488-w

**Published:** 2023-03-14

**Authors:** Hisayuki Nakatani, Yuina Ohshima, Taishi Uchiyama, Suguru Motokucho, Anh Thi Ngoc Dao, Hee-Jin Kim, Mitsuharu Yagi, Yusaku Kyozuka

**Affiliations:** 1 Polymeri Materials Laboratory, Chemistry and Materials Program, Nagasaki University, 1-14 Bunkyo-machi, Nagasaki, 852-8521 Japan; 2 Graduate School of Fisheries and Environmental Sciences, Nagasaki University, 1-14 Bunkyo-machi, Nagasaki, 852-8521 Japan; 3 Organization for Marine Science and Technology, Nagasaki University, 1-14 Bunkyo-machi, Nagasaki, 852-8521 Japan

**Keywords:** Marine chemistry, Environmental chemistry, Green chemistry, Materials chemistry, Polymer chemistry, Environmental sciences, Chemistry

## Abstract

Various tiny plastic particles were retrieved from the sea and studied using scanning electron microscopy/energy-dispersive X-ray spectroscopy (SEM/EDX) analysis to prepare realistic reference microplastics (MP). Most of the MP exhibited a diameter of < 20 × 10^−6^ m and 0.1–0.2 molar ratios of oxygen to carbon atoms (O/C), indicating that they primarily comprised polyethylene (PE), polypropylene (PP), and polystyrene (PS). It took a long time to reproduce such O/C ratios in standard laboratory weathering methods. For example, degrading of 30 × 30 × 0.060 mm PP film required 75 days for the 0.1 ratio, even with an advanced oxidation process (AOP) using a sulfate radical anion (SO_4_·^−^) initiator in distilled water at 65 °C. However, seawater drastically improved the PP degradation performance of AOP under a weak acid condition to achieve the 0.1 ratio of PP film in only 15 days. The combination of seawater and the SO_4_·^−^ initiator accelerated the degradation process and showed that the MP’s size could be controlled according to the degradation time.

## Introduction

A part of plastic litter presents a severe issue when discarded in the marine environment because it spreads to the sea and leads to MP pollution^1–14^. PE, PP, PS, polyvinylchloride (PVC), and polyethylene terephthalate (PET) are produced globally on a huge scale. These commercially produced plastics constitute approximately 80% of all thermoplastics^15,16^. For example, PP and PE represent 22% and 23% of Japanese resin manufacturing (in 2018), respectively. Most plastic litter comprises PE, PP, PS, PVC, and PET^17^. Consequently, marine MP production mainly results from degradation of macroscopic PP, PE, and PS pieces, which are likely generated by sunshine exposure^12–14^. The MPs of PP, PE, and expanded PS (EPS) products float on the sea surface due to low density. These polymers have poor biodegradability and remain in the marine environment for a long time. Furthermore, the MPs partially spread from the sea to the atmosphere^18^. The primary place of MP production has not been clarified yet. For instance, one group was generated in a terrestrial region (on land) and others in the sea^19^. Although the MPs are produced in various places, it is considered that they are primarily produced in the sea and its surroundings. The MP production mechanisms on land and sea differed considerably^19^. Delamination was observed on the surface of MPs retrieved from the seashore. However, no delamination marks but an abrasion patch structure occurred on the MP surface retrieved from the riverside^19^. The delamination part becomes a much smaller MP, which is released into the sea. MP preparation in seawater is desired for a realistic reference sample.

In our previous study, PP degradation tests were performed in distilled water via an advanced oxidation process (AOP) using SO_4_·^−^^19^, where SO_4_·^−^ functioned as a highly efficient initiator for plastic degradation. However, many types of organic and inorganic constituents exist in the sea. Specifically, Cl^−^ reacts with OH· and inhibits photodegradation (autoxidation) initiation^20,21^. Marine MP generation involves autoxidation in seawater. Therefore, to investigate the effects of marine MP on marine ecosystems, it is necessary to develop an accelerated degradation method to establish a realistic reference of marine MP quickly; however, the effects of Cl^−^ inhibiting the autoxidation process make it challenging. Therefore, a new initiator is needed to replace OH· to promote autoxidation in seawater. SO_4_·^−^ would also be an effective initiator in seawater, as Cl^−^ converts it to OH·^22^, with some produced OH· simultaneously inhibited by it. There is competition between the two species; however, the preponderance of the OH· formation promotes the autoxidation process. Furthermore, SO_4_·^−^ is gradually converted to SO_4_^2–^, affecting the pH of alkaline seawater, with the equilibria of the reaction dependent on pH^23^. This change in pH accelerates the autoxidation process. It is considered that SO_4_·^−^ shortens the time required to prepare the realistic reference of marine MP because of these effects.

There is a need for realistic reference material that simulates environmental MPs in the oceans. Therefore, we collected and characterized marine MPs in the sea near Nagasaki Prefecture, Japan. They simulated accelerated marine degradation of PP in the laboratory, and compared the degradation products with the marine MPs. In this study, the size and O/C molar ratios of marine MP particles were determined using scanning electron microscopy (SEM)/energy-dispersive X-ray spectroscopy (EDX) analysis to establish a new method for preparing a realistic reference marine MP. The reference PP was prepared via AOP degradation using SO_4_·^−^ as an autoxidation initiator in seawater. The degraded sample was studied for its degradation behavior, shape, O/C molar ratio, and size to evaluate the action of seawater on the plastic. The obtained results were evaluated for their equivalence with marine MP.

## Materials and methods

### Materials

PP was supplied by Prime Polymer Co., Ltd. (product name: J-700GP). The Melt flow rate (MFR: ISO 1133:2005) and density were 8 g/10 min and 0.9 g/cm^3^, respectively. Potassium persulfate (K_2_S_2_O_8_) and bumetrizole were purchased from Wako Pure Chemical Industries. Sea water was retrieved from Nagasaki fishing port in Nagasaki city, Nagasaki, Japan (around S1 in Table S1).

### Scanning electron microscope (SEM) with energy dispersive x-ray spectroscopy analysis

The SEM/EDX analysis was carried out with a JSM-7500FAM (JEOL) at 5.0 kV. The working distance was about 3 × 4 mm. Samples were placed in dried oven maintained at 27 °C for 30 min and were sputter-coated with gold before SEM imaging.

### Particle size measurement

The fragment sample size was measured with an optical microscope (Nikon ECLIPSE 50/POL) or with a dynamic light scattering method (Otsuka Electronics Co., Ltd. ELSZ-2000ZS).

### Differential scanning calorimetry (DSC) measurement

The DSC measurements were made with a SHIMADZU DSC-60 Plus. The 5 mg samples were sealed in aluminum pans. The measurement of the samples was carried out at a heating rate of 10 °C/min in the measurement range from 30 to 250 °C under a nitrogen atmosphere.

### MP retrieving from the sea

MP retrieving was carried out on 26th July 2021 using the training vessel T/V Kakuyo-maru (155 gross tonnage: Faculty of Fisheries, Nagasaki University)^24,25^. The sampling stations were summarized in Table S1. Each sampling of 1 L sea water was carried out in one day. The samples denoted as “D” were collected using a Conductivity Temperature Depth profiler (CTD) system at ca. 50 m below the sea level (the sampling site was middle of the water column), and on the other hand the surface sampling (denoted as “S”) was carried out with a 3 L stainless bucket. The CTD system comprised with a rosette of Niskin bottles made of polyvinylchloride (PVC) and a CTD profiler. The sampled seawater was transferred to a 1-L bottle made of PE and stored. There were contaminants of PVC and PE MPs as a matter of course. Therefore, particles identified as PVC by SEM/EDX analysis were removed from marine MPs (a few). The contaminants of PE MPs were counted as they were, although they were considered to be coexistent with unoxidized materials (O/C ≈ 0).

### Filtration for SEM/EDX observation of MP retrieved from the sea

Filtration was carried out with a polycarbonate membrane filter (Merck Isopore™ membrane) with 8 µm pore size without pretreatment. The filtration volume per sample was 100 ml, and one filter was used for the filtration of one sample.

### Degradation using advance oxidation process (AOP)

The PP film was molded into thin films (30 × 30 × 0.060 mm) by compression molding at 180 °C under 10 MPa for 11 min. The AOP degradation procedure was according to the previous reports^22,26^. (1) Each five pieces of the film were put into a 100 ml glass vessel equipped with a 20 ml aqueous solution containing 0.54 g K_2_S_2_O_8_ at ca. 65 °C for 12 h under stirring by a stirrer tip with a speed of ca. 100 rpm. (2) The equal amount of K_2_S_2_O_8_ aqueous solution was added to compensate for the consumption of oxidant, and the degradation was continued for another 12 h under the same conditions. (3) And then only the five pieces of the film were moved to a new 100 ml glass vessel equipped with a 20 ml aqueous solution containing 0.54 g K_2_S_2_O_8_, and their AOP degradations were restarted under the same conditions. The AOP degradation was carried out for a predetermined number of days using (1) to (3) as one set. The pH value of solution was changed from 8 to 3 during the one set.

### Preparation of PP sample containing Ultraviolet absorber (UVA)

Bumetrizole was used as an UVA. A PP sample containing 5 phr bumetrizole was prepared by an Imoto Seisakusyo IMC-1884 melting mixer. The mixing was performed at 190 °C and 100 rpm for 5 min and was molded into a thin film (50 × 50 × 0.050 mm) by compression molding at 190 °C under 50 MPa for 5 min. The degradation was performed using the AOP in distilled water, and its condition was similar to the one described above.

## Results and discussion

### Retrieved MP sizes and their O/C molar ratios

Figures S1 and S2 show SEM photographs of MP samples and plankton debris retrieved from seawater, respectively. Their shapes differ considerably, particularly the plankton debris, which is often derived from diatoms and has a unique honeycomb structure, and it is possible to distinguish it by appearance. Furthermore, differences exist in the constituent elements and composition ratios of elements between them. The plankton debris primarily comprises polysaccharides, such as cellulose, and the O/C molecular ratio is high (0.83). In the case of diatoms, silicon is the primary constituent element. Figure S3 shows the SEM photograph and EDX analyses of MP eroded by diatoms. The SEM/EDX analysis reveals that two diatom types or one diatom and coccosphaerales were attached on the MP surfaces. These results suggest that marine MP can be distinguished from phytoplankton using SEM/EDX analysis.

**Figure 1:**
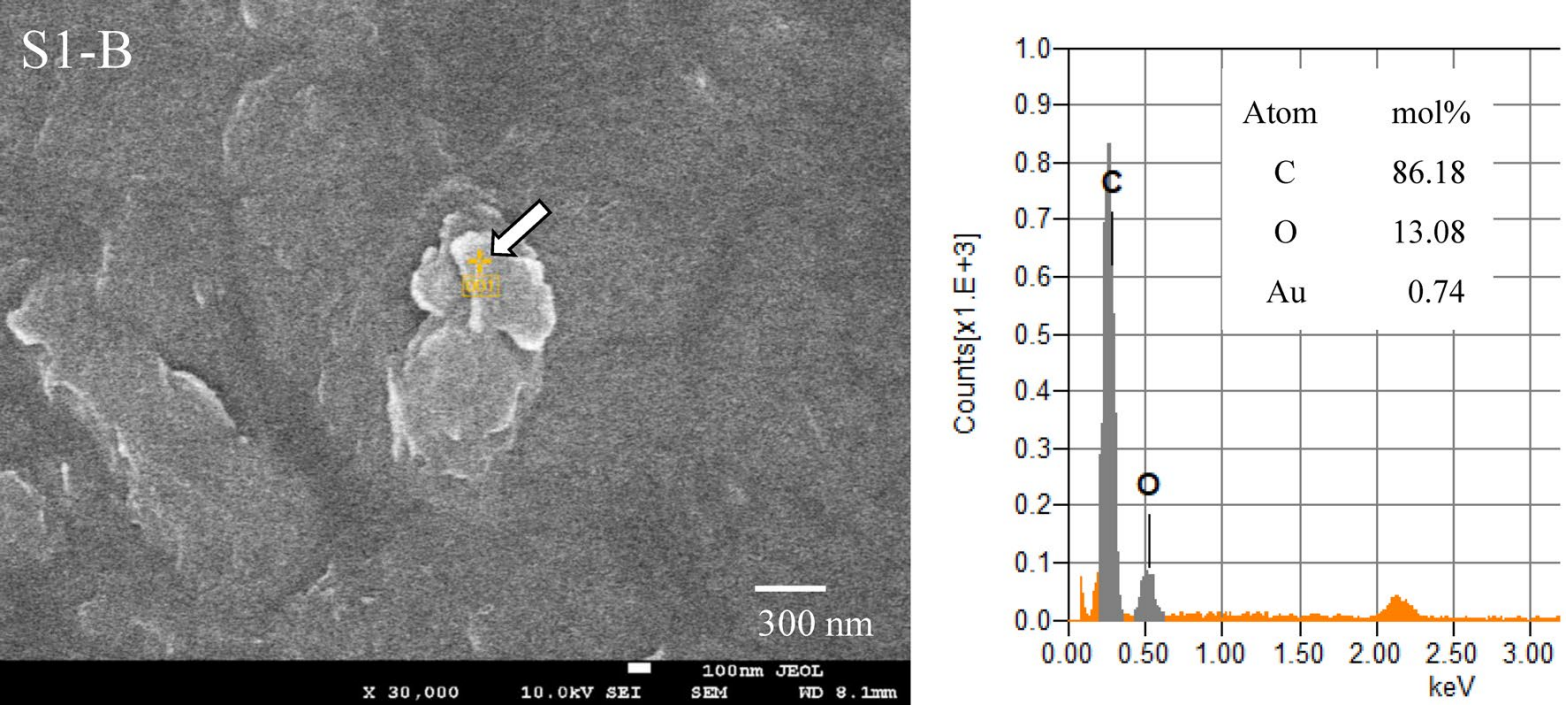
SEM photograph and EDX analysis of a plastic tiny particle sample retrieved by the sea.

**Figure 2:**
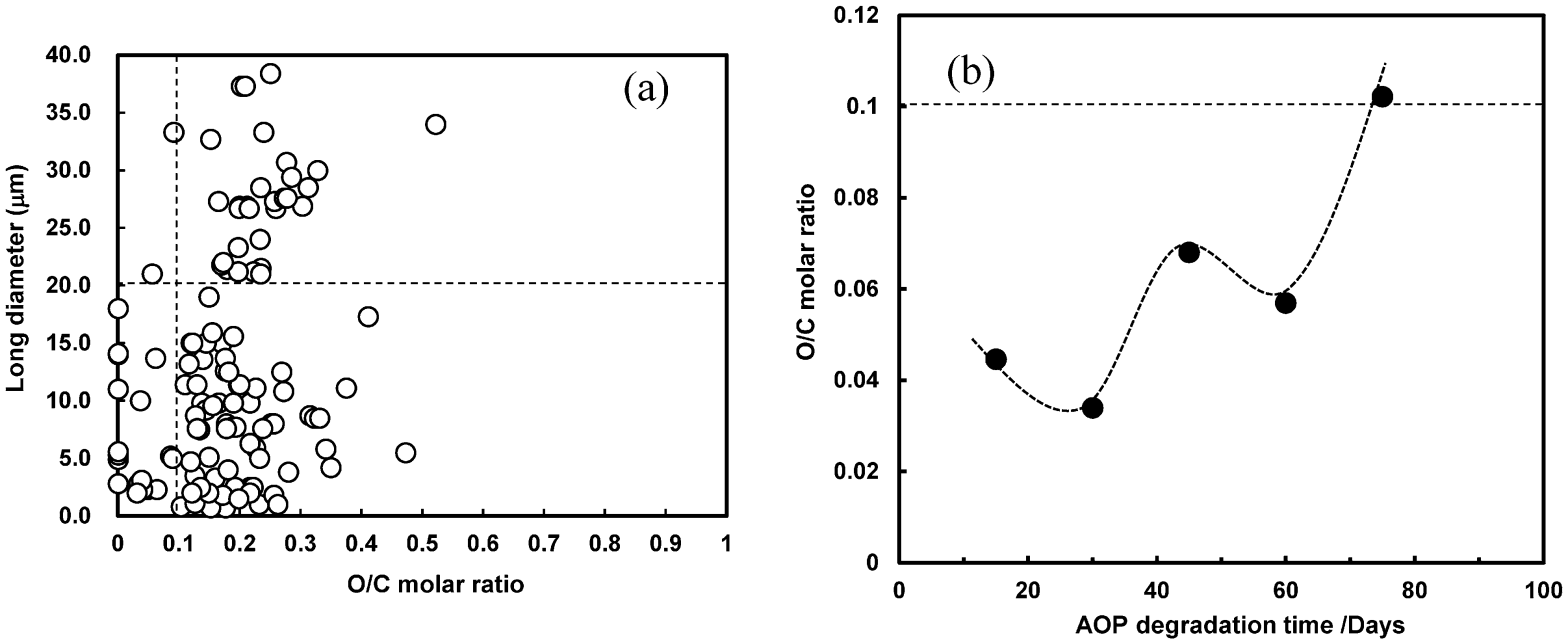
(**a**) Relationship between O/C molar ratio and long diameter (mm) of MP samples retrieved from the sea. (**b**) Relationship between AOP degradation time and O/C molar ratio AOP using a PP sample in distilled water.

Figure 1 shows an SEM image and EDX analysis of a sample of tiny plastic particles retrieved from the sea (sampling station: S1-B), with a particle size of approximately 450 nm and an O/C molar ratio of 0.15. This low ratio indicates that the particles comprise artificial plastic materials. Figure 2a shows the relationship between the O/C molar ratio and the long diameter of MP samples retrieved from the sea. As shown in Fig. 2a, most MP samples show O/C molar ratios of < 0.4. The MP contained almost no PET with the 0.4 O/C molar ratio. In addition, the O/C molar ratios of pure PE, PP and PS not containing oxygen atoms are zero. As shown in Fig. 2a, the number of MP showing the 0 value was several, which accounted for an extremely small percentage of the total, and the number of pure, i.e., undegraded, PE, PP, and PS was small. Similarly, only a small number of the MP was identified as PVC with high chlorine content.

The O/C molar ratios indicate that the MP samples retrieved from the sea primarily comprise PE, PP, and PS. Most MP particles exhibit diameters less than 20 μm long and O/C molar ratios of 0.1 or more (Fig. 2a). These results provide a size index and degradation degree (degree of oxidation) for preparing a realistic reference MP. Plastics typically contain additives, such as UVA, antioxidants. UVA and antioxidants are added to suppress autoxidation, i.e., degradation, which are consumed as the degradation progresses. Thus, the residual amounts of additives decrease as the MPs become smaller due to degradation. Figure S4 shows the relationship between the AOP degradation time and UVA retention ratio using a PP reference sample containing 5 phr UVA (bumetrizole) in distilled water. The UVA retention ratio rapidly decreased with the degradation time, indicating that a real small-sized MP contains little additives, such as UVA and autoxidation.

However, the flame retardants might remain, but the retrieved MPs with high metal oxide concentrations from the EDX analysis were excluded from the plots of Fig. 2a. Brominated flame retardants were hardly contained in the retrieved MPs according to the absence of Br atoms in the EDX results. Consequently, the O/C molar ratios of the retrieved MPs indicate the original composition ratios or oxidation degree on the surfaces. Figure 2b shows the relationship between the AOP degradation time and the O/C molar ratio of a PP sample in distilled water. The ratios gradually increased with up and slight down. This behavior can be attributed to repeated oxidation and delamination^19,27^. After 75 days, an O/C of 0.1 was achieved (Fig. 2b), indicating that it takes a long time to use the AOP degradation method to achieve the same O/C ratio for PP as the degradation level of the retrieved MPs. It is thus necessary to develop a method to quickly prepare reference MPs to investigate their effect on marine organisms.

### Inhibiting and accelerating effects on autoxidation by salinity

**Figure 3:**
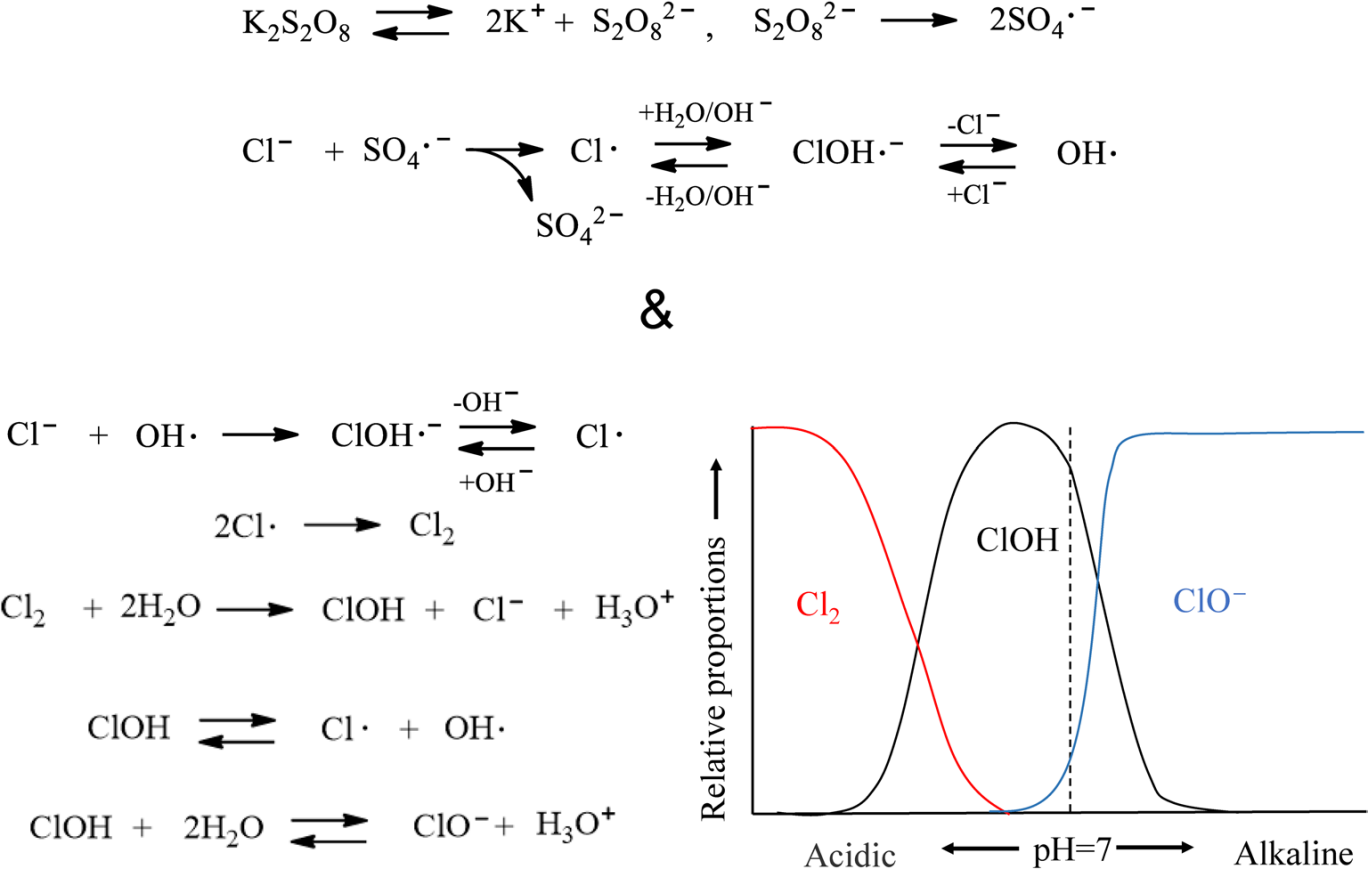
A transformation of radical species from SO_4_·^−^ to OH· in seawater, mechanisms for transfer reaction to Cl^−^ and generation of chemical composition of Cl_2_ solution in function of pH.

Salinity lowers the degradation level of polyolefins, such as PP and PE^21,28,29^. The refractive index of seawater increases due to salinity, and the UV light use rate decreases in the degradation^28,29^. Wu et al. reported that aqueous Cl^−^ functions as an inhibitor in the photooxidation of PP in seawater^21^. Figure 3 shows the transformation of radical species from SO_4_·^−^ to OH· in seawater. For PP photodegradation in seawater, Cl^−^ reacts with OH· generated by solar irradiation and converts to ClOH·^−^, a less reactive molecule^21^. Therefore, it is necessary to change the initiator of the oxidation degradation (autoxidation) reaction from OH· to another radical species to avoid the inhibitory effect of Cl^−^. Considering the reactivity of radical species, SO_4_·^−^ is suitable for autoxidation in seawater. As shown in Fig. 3, the Cl^−^ transforms the SO_4_·^−^ initiator^22^. A large amount of SO_4_·^−^ is converted into OH·, with some of the OH· converted into ClOH·^−^. However, since ClOH·^−^ production requires a re-reaction with Cl^−^, the residual OH· amount increases. The initiation efficiency of autoxidation is significantly improved in seawater due to the OH· reactivity being higher than that of SO_4_·^−^. Moreover, a reaction between Cl^−^ and OH· occurs and produces Cl·. Figure 3 shows that two Cl· atoms couple to produce Cl_2_, which then reacts with H_2_O and forms ClOH, with the equilibria of the two reactions dependent on pH^23^. Since the pH of seawater is approximately 8, the equilibrium is biased toward the less reactive ClO^−^, suppressing the autoxidation of PP in seawater. As the SO_4_·^−^ gradually converts to SO_4_^2−^, the pH value of the K_2_S_2_O_8_ in the seawater solution decreases from ca. 8 to ca. 3 by the time of the daily exchange (see “Materials and methods” Section). This procedure ensures a bias in the ClOH-rich equilibrium^23^ for a period before exchanging a fresh K_2_S_2_O_8_ seawater solution. The ClOH has a longer lifetime^22^ and migrates deeply into the polymer matrix before dissociating into radicals and initiating autoxidation^23^. The autoxidation proceeds from the PP interior and the surface, and the MP formation rate is synergistically accelerated. SO_4_·^−^ generates OH· and ClOH, overcoming the inhibiting effect and accelerating the autoxidation process.

**Figure 4:**
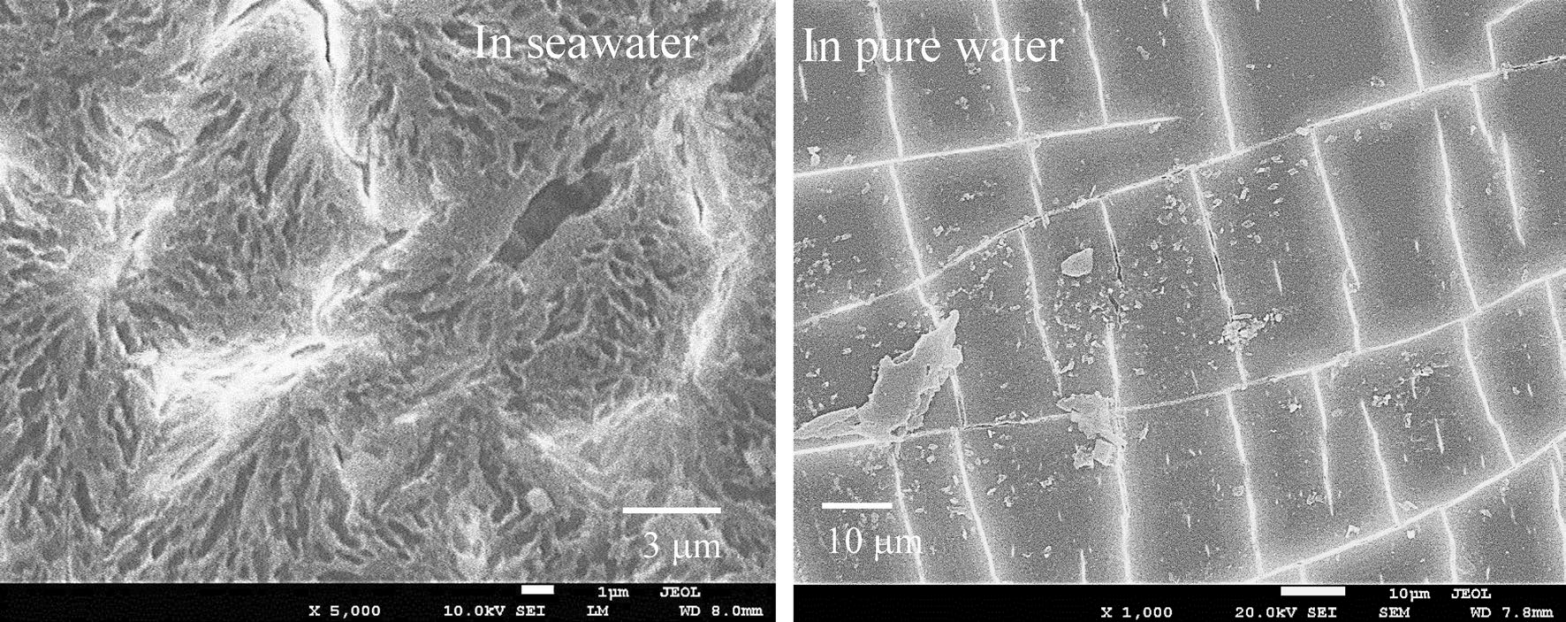
SEM photographs of 15 days-AOP degradation PP in seawater and distilled water.

**Figure 5:**
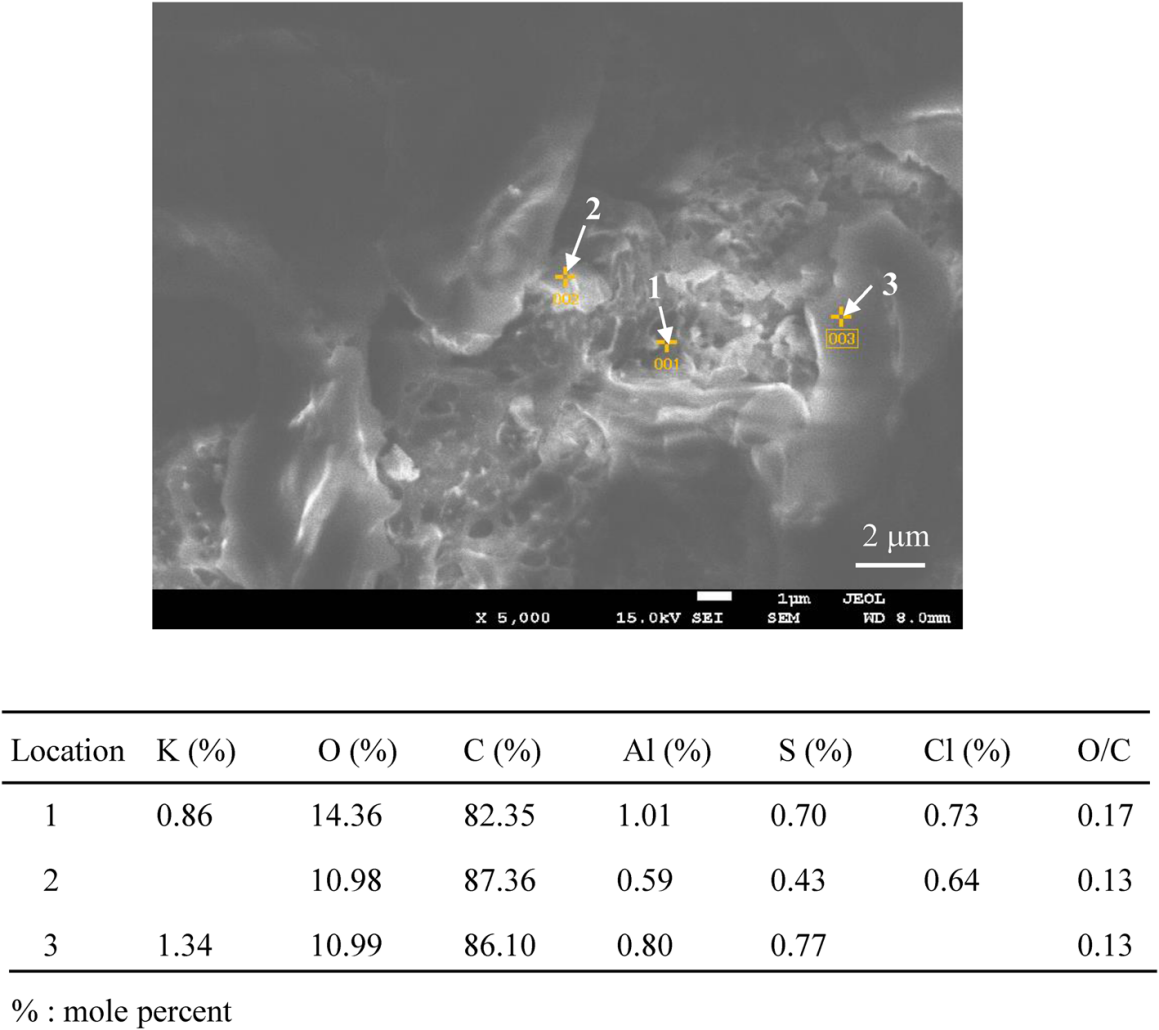
SEM photograph and EDX analysis around delamination on 15 days-AOP degraded PP sample in the seawater.

**Figure 6:**
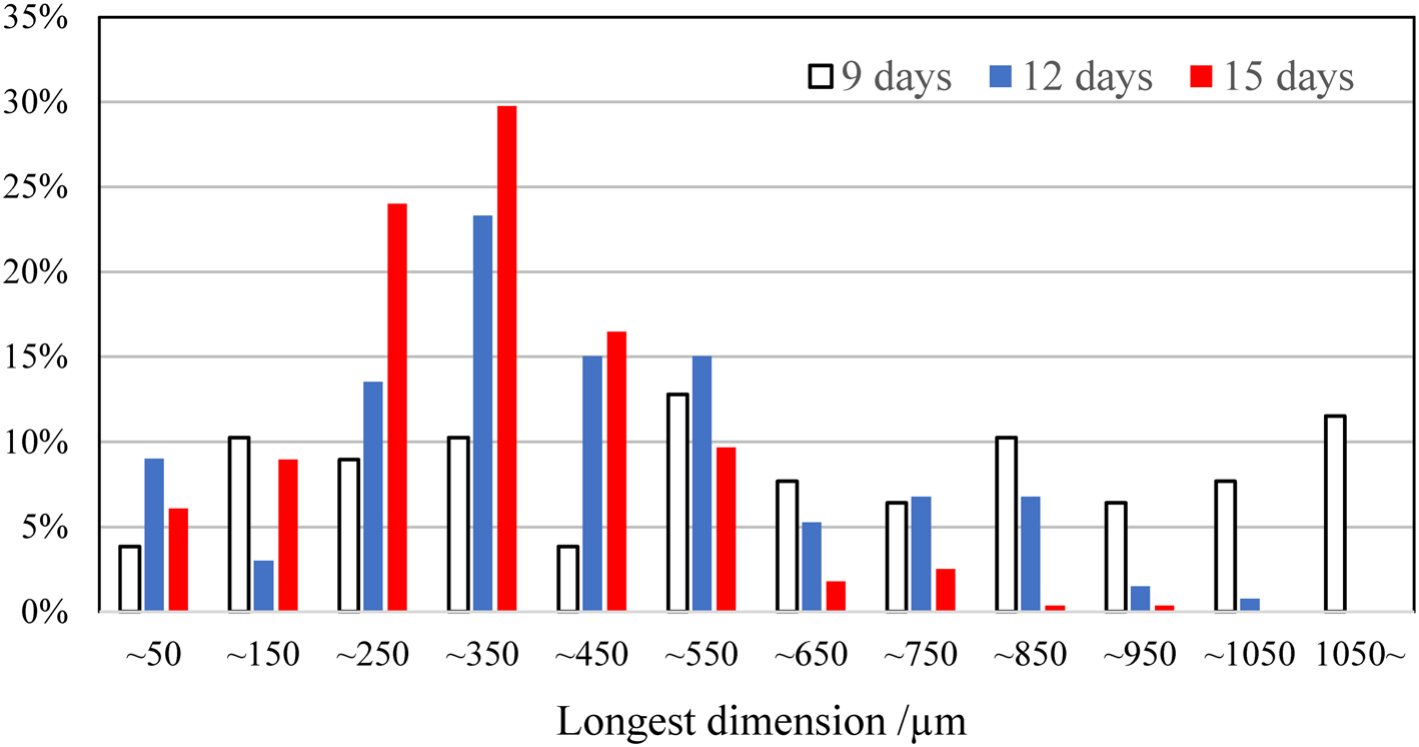
Longest dimension distributions of 9-, 12- and 15 days-AOP degraded PP samples in the seawater. Particle numbers: 9 days = 79, 12 days = 136, 15 days = 279.

**Figure 7:**
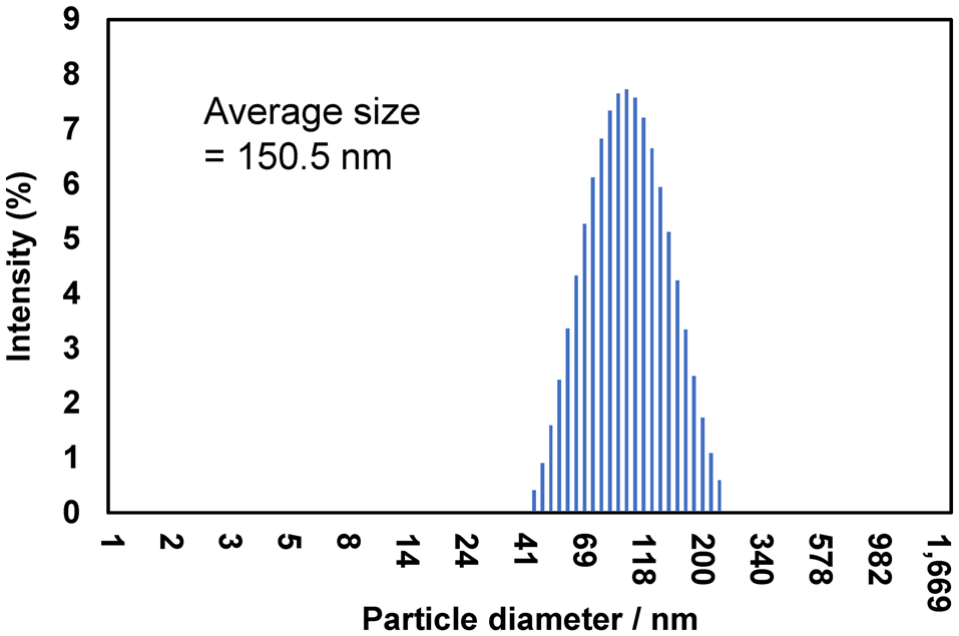
Nanosized particle diameter distribution of 15 days-AOP degraded PP sample in the seawater.

Figure 4 shows SEM images of PP degraded via AOP for 15 days in seawater and distilled water. Numerous micropits can be observed on the seawater sample’s surface, which are traces of chemi-crystallization related to the autoxidation process^30,31^, showing that the PP degradation rapidly progresses. However, the distilled water sample’s surface exhibits a smooth lattice-like texture formed by cracks. The degradation degree of the PP in distilled water is thus considerably less than that of the seawater sample. Figure S5 shows the thermal properties of pristine PP and various AOP-degraded PP samples in seawater. The differential scanning calorimetry curves around the melting point (*T*_m_) change considerably with the increase in AOP degradation time. The *T*_m_ decreases from 163.62 to 144.17 °C, and the crystallinity (*X*_c_) goes from 37.35 to 26.32% between the pristine and the 15-day-degraded PP sample, respectively. The changes indicate that the PP crystalline part is degraded. Although crystalline polymer degradation is frequently limited to the amorphous part, the seawater AOP method degrades the crystalline one. Figure 5 shows SEM images and EDX analysis around the delamination location on the PP sample degraded via AOP for 15 days in seawater. Many micro-sized delamination marks can be observed, and the O/C molar ratios are 0.17, 0.13, and 0.13 (the arrows in Fig. 5). These values are similar to those of the MP samples retrieved from the sea, indicating that an MP sample with the same degradation degree can be prepared in a short degradation time of 15 days. Moreover, combining seawater and a K_2_S_2_O_8_ initiator promotes excellent accelerated plastic degradation. Furthermore, the difference in the degradation degree at the measurement location and between the surface and the interior is small. The permeability of the degradation initiator is high, and it would not be easily affected by the difference in the medium. In our previous study, a PP film degradation test was performed in water using a specific photocatalyst under visible light irradiation^27^. The result revealed that planar exfoliation via autoxidation in water generated MPs, and the MP size depended on the degradation degree, i.e., the degradation time. Figure 6 shows the longest dimension distributions of PP degraded via AOP over 9, 12, and 15 days. After 9, 12, and 15 days of degradation, 79, 136, and 279 MP particles were recovered by filtration, respectively, indicating that the size depends on the AOP degradation time as well. The size distribution narrows with increasing AOP degradation time, with a bias toward smaller sizes. Figure 7 confirms that nanosized PP particles are obtained over 15 days of AOP degradation, indicating that the MP size can be controlled according to the degradation time.

## Conclusion

To establish preparation of realistic reference MP, many MPs retrieved from the sea were measured in terms of their sizes and O/C molar ratios. Most of them had a size of < 20 μm in diameter and O/C molar ratios of 0.1–0.2. It was indicated that the reference MP should be made of PE, PP and PS from the low O/C ratio. After 75 days of the AOP degradation of PP in distilled water, an O/C of 0.1 was reached. It took a long time for the O/C ratio of the AOP degradation of PP in water using a SO_4_·^−^ initiator in water to reach the same value as that of the marine MP. However, the SO_4_·^−^ initiator was converted into OH· by aqueous Cl^−^ in seawater, and the initiation efficiency was greatly improved in seawater. Moreover, the SO_4_·^−^ gradually converted to SO_4_^2−^ and led to a bias toward a ClOH-rich equilibrium. The ClOH dissociated into OH·. The use of SO_4_·^−^ overcame the Cl^−^ inhibiting effect on the autoxidation process and provided an accelerating effect in seawater. The O/C molar ratio of the PP sample degraded via AOP over 15 days in seawater was the same as that of the marine MP. The combination of seawater and K_2_S_2_O_8_ initiator thus promoted excellent accelerated degradation.

Many micro-sized delamination marks were observed for the PP sample degraded via AOP over 15 days in seawater, with O/C molar ratios of around 0.17, 0.13, and 0.13 measured from different positions. These values are similar to those of MP samples retrieved from the sea, indicating that an MP sample with the same degree of degradation of the sea-derived MP can be prepared in a short degradation time of 15 days. The combination of seawater and SO_4_·^−^ initiator promoted excellent accelerated degradation of the plastic. Nanosized PP particles were obtained over 15 days of AOP degradation, showing that the size of MP could be controlled according to the degradation time.

## Supplementary Information

Supplementary Figure S1.

Supplementary Figure S2.

Supplementary Figure S3.

Supplementary Figure S4.

Supplementary Figure S5.

Supplementary Table S1.

## Data Availability

The data that support the findings of this study are available from the corresponding author, [HN], upon reasonable request.

## References

[1] Derraik JGB (2002). The pollution of the marine environment by plastic debris: A review. Mar. Poll. Bull..

[2] Barnes DKA, Galgani F, Thompson RC, Barlaz M (2009). Accumulation and fragmentation of plastic debris in global environments. Philos. Trans. R. Soc. B.

[3] Thompson RC, Swan SH, Moore CJ, vom Saal FS (2009). Our plastic age. P Philos. Trans. R. Soc. B.

[4] Andrady AL (2011). Microplastics in the marine environment. Mar. Pollut. Bull..

[5] Jambeck JR, Geyer R, Wilcox C, Siegler TR, Perryman M, Andrady A, Narayan R, Law KL (2015). Plastic waste inputs from land into the ocean. Science.

[6] Halle AT, Ladirat L, Gendre X, Goudouneche D, Pusineri C, Routaboul C, Tenailleau C, Duployer B, Perez E (2016). Understanding the fragmentation pattern of marine plastic debris. Environ. Sci. Technol..

[7] Avio CG, Gorbi S, Regoli F (2017). Plastics and microplastics in the oceans, from emerging pollutants to emerged threat. Mar. Environ. Res..

[8] Yokota K, Waterfield H, Hastings C, Davidson E, Kwietniewski E, Wells B (2017). Finding the missing piece of the aquatic plastic pollution puzzle, Interaction between primary producers and microplastics. Limnol. Oceanogr. Lett..

[9] Rummel CD, Jahnke A, Gorokhova E, Kühnel D, Schmitt-Jansen M (2017). Impacts of biofilm formation on the fate and potential effects of microplastic in the aquatic environment. Environ. Sci. Technol. Lett..

[10] Law KL (2017). Plastics in the marine environment. Annu. Rev. Mar. Sci..

[11] Michels J, Stippkugel A, Lenz M, Wirtz K, Engel A (2018). Rapid aggregation of biofilm-covered microplastics with marine biogenic particles. Proc. R. Soc. B.

[12] Lambert S, Wagner M (2016). Formation of microscopic particles during the degradation of different polymers. Chemosphere.

[13] Julienne F, Delorme N, Lagarde F (2019). From macroplastics to microplastics: Role of water in the fragmentation of polyethylene. Chemosphere.

[14] Julienne F, Lagarde F, Delorme N (2019). Influence of the crystalline structure on the fragmentation of weathered polyolefines. Polym. Degrad. Stab..

[15] Plastics Europe. *Plastics-The Facts 2019* (2019).

[16] Gerritse J, Leslie HA, de Tender CA, Devriese LI, Vethaak AD (2020). Fragmentation of plastic objects in a laboratory seawater microcosm. Sci. Rep..

[17] Gewert B, Plassmann MM, MacLeod M (2015). Pathways for degradation of plastic polymers foating in the marine environment. Environ. Sci. Process. Impacts.

[18] Allen S, Allen D, Moss K, Le Roux G, Phoenix VR, Sonke JE (2020). Examination of the ocean as a source for atmospheric microplastics. PLoS ONE.

[19] Nakatani H, Muraoka T, Ohshima Y, Motokucho S (2021). Difference in polypropylene fragmentation mechanism between marine and terrestrial regions. SN Appl. Sci..

[20] Grebel J, Pignatello J, Mitch W (2010). Effect of halide ions and carbonates on organic contaminant degradation by hydroxyl radical-based advanced oxidation processes in saline waters. Environ. Sci. Technol..

[21] Wu X, Liu P, Wang H, Huang H, Shi Y, Yang C, Gao S (2021). Photo aging of polypropylene microplastics in estuary water and coastal seawater: Important role of chlorine ion. Water Res..

[22] Lee J, von Gunten U, Kim JH (2020). Persulfate-Based advanced oxidation, critical assessment of opportunities and roadblocks. Environ. Sci. Technol..

[23] Mikdam A, Colina X, Minard G, Billon N, Maurin R (2017). A kinetic model for predicting the oxidative degradation of additive free polyethylene in bleach desinfected water. Polym. Degrad. Stab..

[24] Kobayashi T, Yagi M, Kawaguchi T, Hata T, Shimizu K (2021). Spatiotemporal variations of surface water microplastics near Kyushu, Japan: A quali-quantitative analysis. Mar. Pollut. Bull..

[25] Yagi M, Kobayashi T, Maruyama Y, Hoshina S, Masumi S, Aizawa I, Uchida J, Kinoshita T, Yamawaki N, Aoshima T, Mori Y, Shimizu K (2022). Microplastic pollution of commercial fishes from coastal and offshore waters in southwestern Japan. Mar. Pollut. Bull..

[26] Liu P, Qian L, Wang H, Zhan X, Lu K, Gu C, Gao S (2019). New insights into the aging behavior of microplastics accelerated by advanced oxidation processes. Environ. Sci. Technol..

[27] Nakatani H, Kyan T, Muraoka T (2020). An effect of water presence on surface exfoliation of polypropylene film initiated by photodegradation. J. Polym. Environ..

[28] Quan X, Fry ES (1995). Empirical equation for the index of refraction of seawater. Appl. Opt..

[29] Cai L, Wang J, Peng J, Wu Z, Tan X (2018). Observation of the degradation of three types of plastic pellets exposed to UV irradiation in three different environments. Sci. Total Environ..

[30] Rabello MS, White JR (1997). Crystallization and melting behaviour of photodegraded polypropylene—I. Chemi-crystallization. Polymer.

[31] Craig IH, White JR, Kin PC (2005). Crystallization and chemi-crystallization of recycled photo-degraded polypropylene. Polymer.

