# Supplementary figures and images for: Rapid oxidative fragmentation of polypropylene with pH control in seawater for preparation of realistic reference microplastics
Source: Sci Rep. 2023 Mar 14;13:4247. doi: 10.1038/s41598-023-31488-w (PMC10015029; doi:10.1038/s41598-023-31488-w)

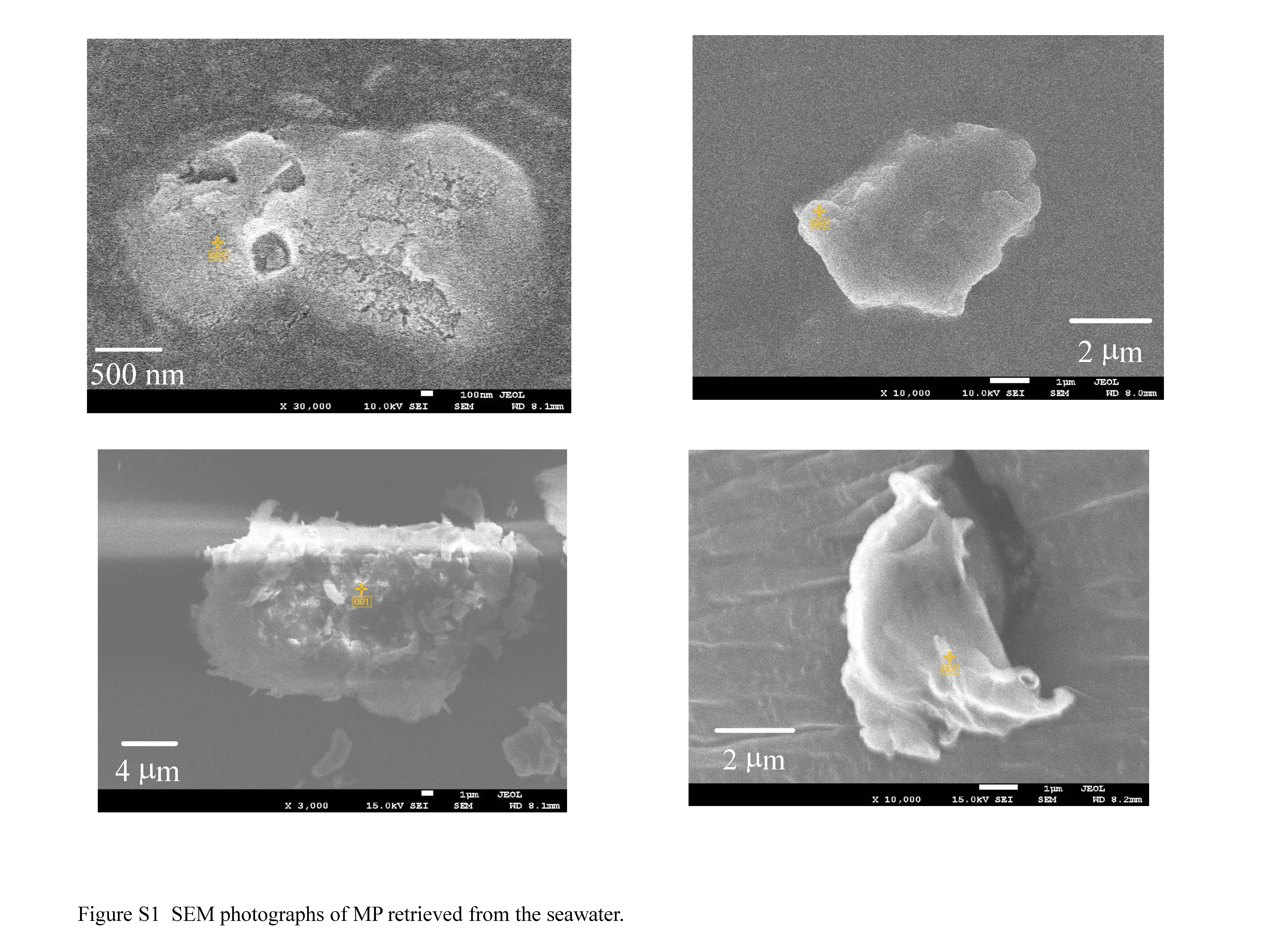

Supplement: Supplementary file 1 — Supplementary Figure S1. [file 41598_2023_31488_MOESM1_ESM.tiff]

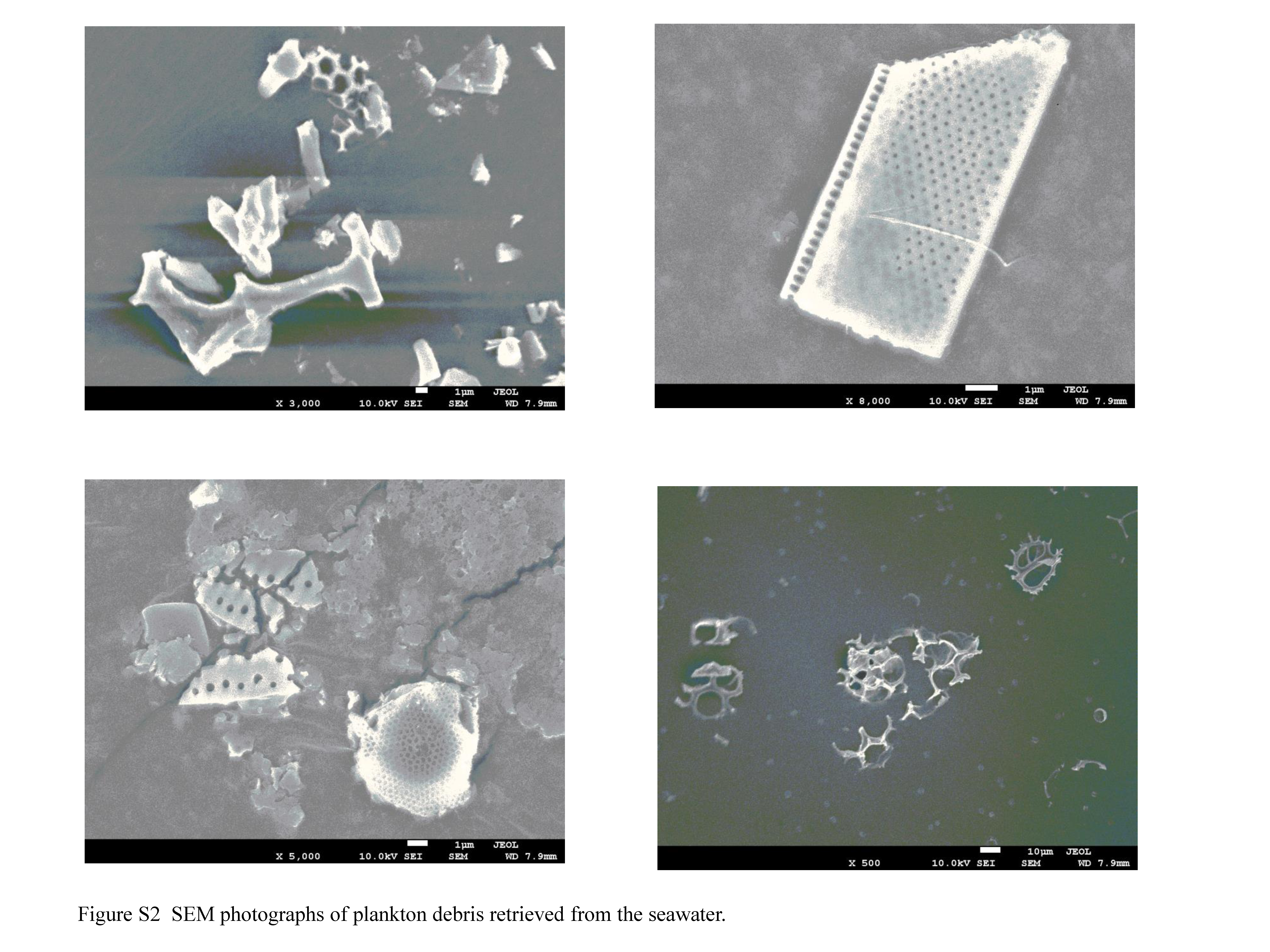

Supplement: Supplementary file 2 — Supplementary Figure S2. [file 41598_2023_31488_MOESM2_ESM.tiff]

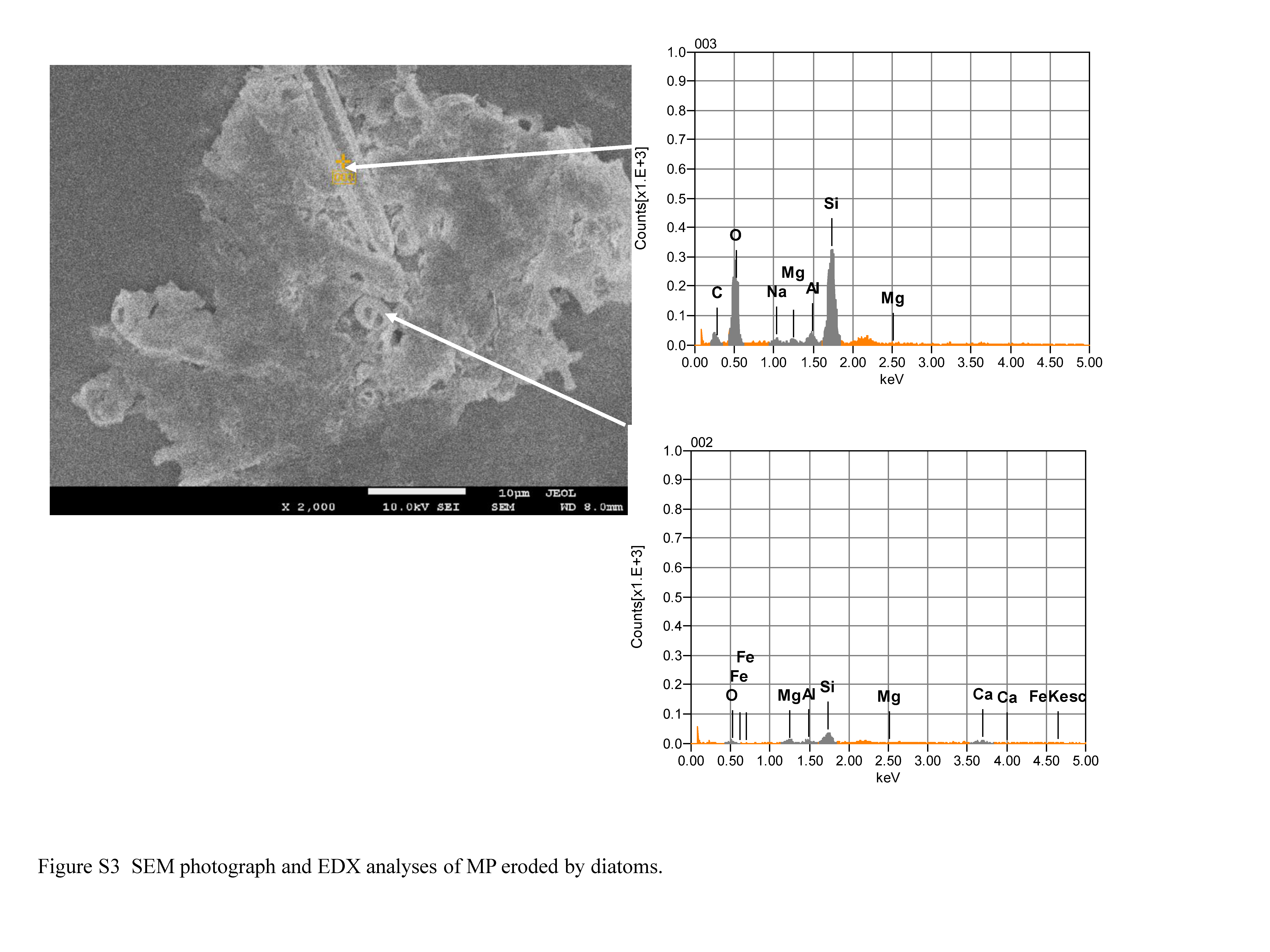

Supplement: Supplementary file 3 — Supplementary Figure S3. [file 41598_2023_31488_MOESM3_ESM.tiff]

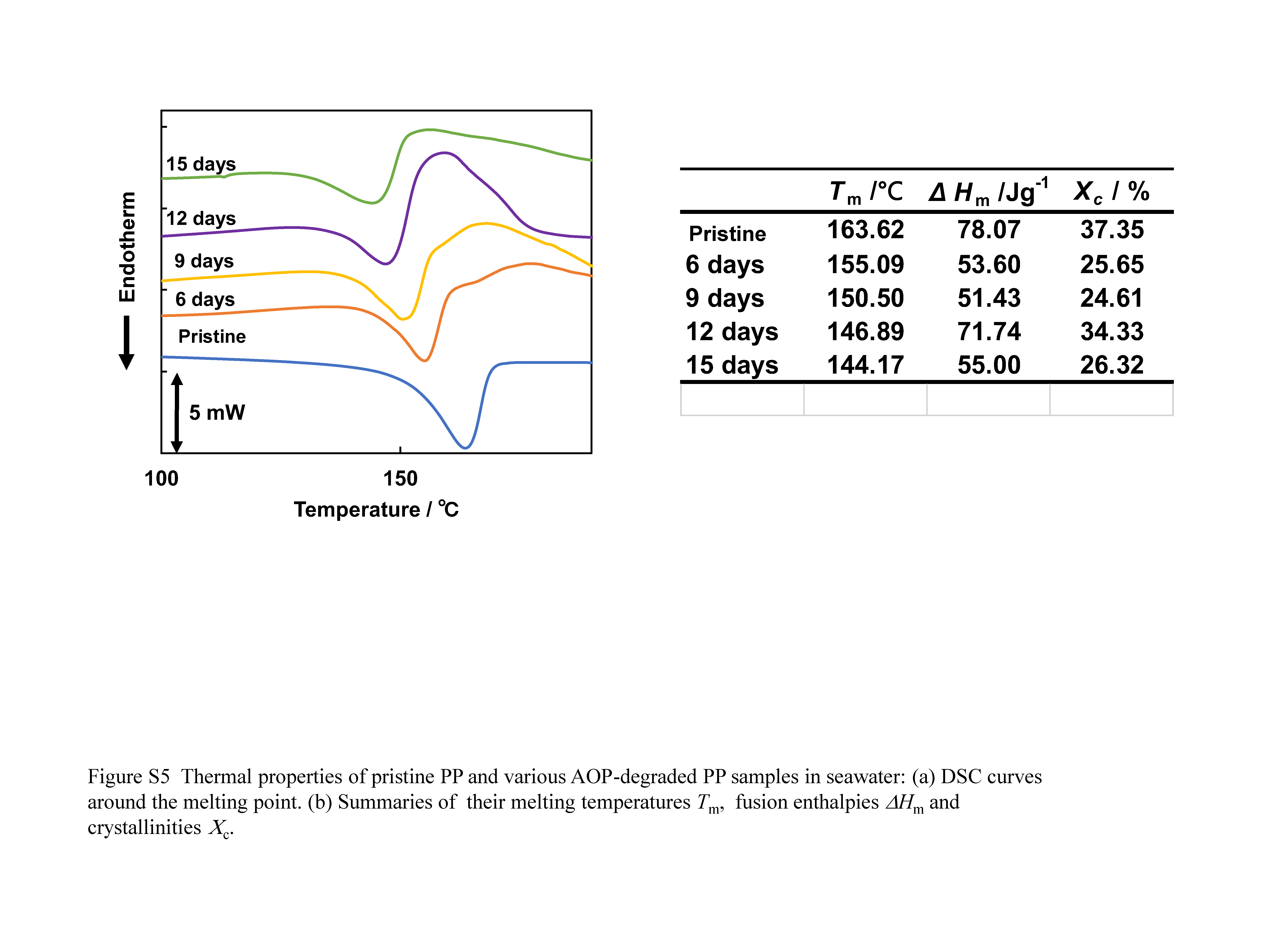

Supplement: Supplementary file 5 — Supplementary Figure S5. [file 41598_2023_31488_MOESM5_ESM.tiff]
