# Supplementary material for: Rapid oxidative fragmentation of polypropylene with pH control in seawater for preparation of realistic reference microplastics
Source: Sci Rep. 2023 Mar 14;13:4247. doi: 10.1038/s41598-023-31488-w (PMC10015029; doi:10.1038/s41598-023-31488-w)
Supplement: Supplementary file 6 — Supplementary Table S1. [file 41598_2023_31488_MOESM6_ESM.docx]

Table S1 Sampling station, coordinates, and depth.

.

Sampling station

Longitude

Latitude

Depth

(m)

0

50

0

50

0

50

0

50

129° 43ʹ 23ʺ E

129° 43ʹ 23ʺ E

129° 24ʹ 17ʺ E

129° 24ʹ 17ʺ E

129° 13ʹ 01ʺ E

129° 13ʹ 01ʺ E

129° 3ʹ 16ʺ E

129° 3ʹ 16ʺ E

32° 46ʹ 49ʺ N

32° 46ʹ 49ʺ N

32° 46ʹ 49ʺ N

32° 46ʹ 49ʺ N

32° 46ʹ 49ʺ N

32° 46ʹ 49ʺ N

32° 46ʹ 49ʺ N

32° 46ʹ 49ʺ N

S1-B

S1-D

S5-B

S5-D

S7-B

S7-D

S9-B

S9-D
